# Autologous Platelet-Rich Plasma Treatment Enables Pregnancy for a Woman in Premature Menopause

**DOI:** 10.3390/jcm8010001

**Published:** 2018-12-20

**Authors:** Konstantinos Sfakianoudis, Mara Simopoulou, Nikolaos Nitsos, Anna Rapani, Athanasios Pappas, Agni Pantou, Margarita Chronopoulou, Efthymios Deligeoroglou, Michael Koutsilieris, Konstantinos Pantos

**Affiliations:** 1 Centre for Human Reproduction, Genesis Athens Clinic, 14-16, Papanikoli, 15232 Athens, Greece; sfakianosc@yahoo.gr (K.S.); nitsos@otenet.gr (N.N.); a_pappas@hotmail.com (A.P.); agnipantos@gmail.com (A.P.); margaritagenesis@gmail.com (M.C.); info@pantos.gr (K.P.); 2 Department of Physiology, Medical School, National and Kapodistrian University of Athens, 75, Mikras Asias, 11527 Athens, Greece; rapanianna@gmail.com (A.R.); mkoutsil@med.uoa.gr (M.K.); 3 Assisted Conception Unit, 2nd Department of Obstetrics and Gynecology, Aretaieion Hospital, Medical School, National and Kapodistrian University of Athens, 76, Vasilisis Sofias Avenue, 11528 Athens, Greece; edeligeo@aretaieio.uoa.gr

**Keywords:** premature menopause, autologous platelet rich plasma, pregnancy, rejuvenation, menstrual restoration

## Abstract

This report presents the case of a woman aged 40 who has experienced premature menopause from the age of 35. Having rejected oocyte donation, she opted for intraovarian injection of autologous platelet-rich plasma with the aim to rejuvenate the ovarian tissue and enable the employment of her own gametes through in-vitro fertilization. Six weeks following the autologous platelet-rich plasma treatment, a significant reduction in the patient’s follicle-stimulating hormone (FSH) levels were noted. A natural in-vitro fertilization cycle led to a biochemical pregnancy, resulting in a spontaneous abortion at the 5th week of pregnancy. This is the first report of a successful autologous platelet-rich plasma application leading to pregnancy in menopause. This report uniquely contributes to the medical knowledge and challenges current practice in the context of infertility. The efficiency and safety of this treatment with regard to the reproductive system merits further investigation.

## 1. Introduction

The complexity of the body’s physiology is reflected in the mechanisms involved in the reproductive system. The leading factor of the female reproductive clock is considered to be age. The menopausal transition experienced after the age of 40 is a phase defined by menstrual cycle irregularities and the fluctuation of hormonal levels, concluded with the final termination of the menstrual period [1]. However, 0.3 to 1.1% of women may experience this spontaneous transition in their reproductive journey prior to the age of 40 [2], classifying them in a state described as premature menopause. Besides the infertility complications concurring with this issue, various medical conditions, such as heart disease and osteoporosis, should be considered [3]. The age of 40 to 45 has been acknowledged to be an established cut-off timeframe for premature menopause and premature ovarian failure, with fluctuations. In this case report we consider premature menopause as the most apposite term, as our patient’s clinical picture and the absence of menstrual cycle portrays an irreversible and final ovarian failure. 

The main characteristic distinguishing patients in premature menopause is amenorrhea. However, practitioners have failed to establish a common consensus regarding the optimal timing to initiate investigations following the absence of the first menstrual cycle in younger patients. It is suggested that the absence of three menstrual cycles should be a valid indication suggesting the investigation of premature menopause [4]. It is important to acknowledge that the majority of patients in this category may report a normal reproductive history, accompanied with regular menses and healthy development in puberty [5], which renders the onset unpredictable. Examining the levels of circulating gonadotropins provides an adequate method for diagnosis. High follicle-stimulating hormone (FSH) levels over 20 mIU/mL accompanied by levels of estradiol (E2) below 30 pg/mL evaluated twice, at an interval of 4 to 6 weeks apart, institute a solid indication of the ovarian dysfunction labeled premature menopause [4].

Thorough consultation is essential in cases where infertility becomes an issue that prematurely menopausal patients wish to address. Patients that are faced with the reality of irregular menstrual cycles or early stages of menstruation absence make up a considerable percentage of patients engaging in the option of assisted reproduction to preserve their fertility through oocyte cryopreservation or in-vitro fertilization (IVF) treatment [4]. In addition to the above, there are cases of patients already in the process of infertility investigation, or already in the process of receiving IVF treatment, who may face premature menopause, premature ovarian failure (POF) or premature ovarian insufficiency (POI). These patients constitute the most challenging cases to manage in order to promote successful infertility treatment. However, in cases of established premature menopause, oocyte donation or adoption may present the only options available. These options are not always welcomed by patients who regularly express their desire to investigate all possibilities to use their own gametes.

Platelet-rich plasma (PRP) is a current trend surfacing in the latest published literature. Studies employing animal models in the reproductive context have reported that PRP enhances the development of primordial and primary preantral follicles [6]. Furthermore, PRP has proven efficacious in preventing possible ischemia following ovarian injury [7]. It is already evident that the employment of PRP to target various issues regarding the reproductive system could be beneficial [8,9,10,11]. PRP institutes an autologous and highly concentrated solution of plasma, which is prepared from the patient’s own blood and contains a concentrated source of growth factors, namely insulin-like growth factor-1 and 2 (IGF-1, IGF-2), fibroblast growth factor (FGF), epidermal growth factor (EGF), transforming growth factor beta (TGF-b), hormones, and cytokines [12]. Considering the active factors involved, along with their potent therapeutic nature, it is not unreasonable to hypothesize that PRP treatment may assist in tissue regeneration [13], the enhancement of anabolic signalling pathways [14], cell differentiation and proliferation [15], angiogenesis initiation, and control [16]. Considering the angiogenic composition of the ovary and the pivotal influence of platelet-derived growth factors on vascular activation and stabilization, treatment with autologous PRP may be viewed as the enabler of ovarian tissue regeneration [17]. PRP contains a member of the TGF-b superfamily, growth differentiation factor 9 (GDF-9) [18]. The *gdf-9* gene expression is regarded as a biomarker of oocyte maturation potential [19,20], and its mutations have been linked to premature ovarian failure [21]. This finding may constitute yet another piece in the completion of the puzzle regarding delineating the basis of the therapeutic potential behind PRP. All this data (from in-vitro studies and animal models to case reports and future trials featuring large datasets) is essential to making new connections and progressing from a hypothesis, to a theory, and to validation enabled by randomized controlled trials (RCTs). The application of PRP in the context of reproductive medicine was pioneered by our team of experts [22], who possess the required background of expertise to approach such a novel trend. In this case report, we describe a singular treatment of a prematurely menopausal patient with the intraovarian injection of autologous PRP. The motivation behind this approach was to initially assess the value of further investigating the hypothesis that PRP implementation could contribute towards partially restoring ovarian function in prematurely menopausal patients. 

## 2. Case Report

Our patient, aged 40, was diagnosed as being prematurely menopausal at the age of 35 following the absence of menstruation for 19 months. During the first appointment, a detailed reproductive examination was performed, including an ultrasound and a hormonal profile assessment. Prior to entering menopause, the patient reported 1 year of failing to achieve natural conception. Unexplained infertility for the couple was diagnosed following an initial infertility investigation. Following the amenorrhea stage, the patient embarked on intermittent hormone therapy (HT) for 3 years and was exploring the possibility of pursuing a pregnancy through IVF treatment. HT was abandoned for a period of 4 months before she was referred to our clinic. The patient’s overall medical record was free of any medical complications. The FSH levels were recorded at 149 mIU/mL and the anti-Müllerian hormone (AMH) levels were 0.02 ng/mL. FSH levels were assessed by chemiluminescent microparticle immunoassay on a Roche Cobas E-411 Immunoassay analyzer. The inter-assay coefficient of variation was <4.6%. AMH levels were assessed at the same time point using the AMH PLUS chemiluminescent microparticle immunoassay on a Roche Cobas E-411 Immunoassay analyzer (Roche Diagnostics GmbH, Mannheim, Germany). 

As protocol dictates, initially the patient was recommended the option of oocyte donation. This option was rejected and the couple expressed their desire to pursue alternative approaches. The current literature cements the therapeutic nature of PRP for various systems [23,24,25], including the reproductive system [25]. In-vitro studies [26], coupled with the numerous registered clinical PRP trials, prompted us to suggest PRP treatment, especially on the grounds that it involves an autologous, minimally invasive, in-house, and complication-free approach. Furthermore, PRP treatment has provided favorable results in our clinic for poor responder patients [27] and patients with chronic endometritis. Following thorough consultation, the patient opted for the PRP approach. The patient provided informed consent prior to participating in the study. The study was conducted in accordance with the Declaration of Helsinki and the protocol was approved by the ethics board of Genesis Athens Clinic (184/6-9-2018). 

The RegenACR^®^-C Kit (Regen Laboratory, Le Mont-sur-Lausanne, Switzerland) was employed to prepare the PRP, which was subsequently injected into the ovaries. The volume of peripheral blood required to prepare 8 mL of PRP for administration was 60 mL. The initial concentration of platelets in the peripheral blood sample was 250,000/µL, while the prepared PRP presented with a concentration of 900,000/µL. According to our protocol, the prepared PRP can be stored at a temperature of 4 °C for a maximum of 1 h. This allows an extent of flexibility regarding the timing of administration that may be required in the clinical setting of a large IVF program. Regarding this case, immediate administration of PRP following preparation was possible and hence we proceeded as such. A volume of approximately 4 mL per ovary was employed for the intraovarian injection. As menopausal women present with ovaries of reduced volume, part of the prepared PRP ultimately resulted in the peritoneal area. The essential parts of the technique consisted of a non-surgical, transvaginal, ultrasound-guided, multifocal, intramedullary injection, and diffusion in the subcortical layers. The injection included multiple sites and, therefore, three punctures were performed per ovary. A 17-gauge needle was employed during the procedure. Restoration of the menstrual cycle was reported 6 weeks following PRP application. The recorded FSH levels were 27 mIU/mL and the AMH levels were 0.08 ng/mL. Employing a natural IVF cycle, not including the use of an antagonist, the patient was subjected to an oocyte collection procedure 8 weeks following PRP administration, specifically 16 days following the reappearance of menstruation. The patient’s follicular growth was assessed via ultrasonography, and hormonal levels were monitored on days 8, 11, and 14 of the menstrual cycle. The follicular diameter was recorded at 11 mm on day 8, 14 mm on day 11, and 18 mm on day 14. Levels of luteinizing hormone (LH) were assessed and recorded at 6.5 mIU/mL, 12 mIU/mL, and 17 mIU/mL on days 8, 11, and 14, respectively. Oestradiol (E_2_) levels were similarly evaluated and recorded at 105 pg/mL, 160 pg/mL, and 270 pg/mL on days 8, 11, and 14, respectively. Lastly, progesterone (PRG) levels were evaluated on day 14 and recorded at 1.4 ng/mL. Following the administration of human chorionic gonadotrophin (hCG) on day 14, oocyte retrieval was enabled 36 h later. The mature oocyte was subsequently inseminated employing intracytoplasmic sperm injection (ICSI). A normally fertilized zygote with two pronuclei led to a six-cell cleavage stage embryo of average grade three quality, according to the morphology grading criteria developed by Veeck and colleagues [28]. Embryo transfer was performed using ultrasound guidance and a 23 cm soft-pass embryo replacement catheter with an echogenic tip. The luteal support scheme included one tablet of cyclacur (estradiol and levonorgestrel), every 8 h per os (oral administration of medication), and vaginally dosed progestin (200 mg) three times per day. Following the embryo transfer, a biochemical pregnancy ensued and was confirmed by a positive human chorionic gonadotropin (hCG) level of 42 mIU/mL. Subsequent hCG measurements were 102 mIU/mL and 800 mIU/mL. Unfortunately, at week 5 of gestation the patient reported a spontaneous abortion. 

## 3. Discussion

This case report describes a prematurely menopausal woman presenting at our clinic with the urge to explore alternative approaches—excluding oocyte donation—to enable her to overcome the reproductive barrier of premature menopause. The option of engaging in PRP treatment was presented to the patient based on the published literature and our recent clinical experience with poor responders that indicated promising results [27]. Various models reported in published studies describe PRP as an effective tool for the regeneration of the reproductive system. Ovarian regeneration was revealed by studying ovarian torsion in a rat model where PRP diminished the high total oxidant status and the ovarian histopathological scores, while also significantly increasing the peritoneal vascular endothelial growth factor levels [7]. In humans, PRP has been employed in an autologous ovarian transplantation in order to improve the quality and the vascularization potential of the implant [29]. Intrauterine PRP treatment has also been proposed to promote endometrial growth in patients with a thin endometrium, improving the assisted reproductive outcome [8]. 

Given the highly angiogenic structure of the ovary and the critical role of various platelet-derived factors in vascular activation and stabilization [30], PRP application is proposed to have enriched the dysfunctional and prematurely menopausal ovarian tissue of our patient with essential factors for neoangiogenesis. In addition, the detection of ovarian stem cells (OSCs) or germline stem cells (GSCs) [19], and embryonic-like stem cells (VSEL) [31,32] in human ovarian surface epithelium—even of post-menopausal and POF women [32]—and their ability to differentiate into oocytes under certain conditions, create new data for the origin of PRP-derived follicles. The significantly decreased levels of FSH following PRP treatment constituted an improvement in regard to the overall reproductive potential of our patient. Thus, this interesting outcome could present an indicative marker of the therapeutic nature of PRP application. On another note, the authors refrain from any statements regarding the slightly increased AMH levels, since the interpretation of the fluctuating AMH levels—even during the menstrual cycle—is often described as conflicting in the literature [33]. PRP’s long-term effectiveness, and serial measurements of FSH and AMH levels following PRP application should be further evaluated in the context of a randomized control trial (RCT). 

Although it is challengingto define with certainty the trail of molecular and physiological events leading to the folliculogenesis, along with the menstrual cycle restoration our patient experienced, it is clear the PRP treatment triggered and enabled this cascade of events. Ovarian primordial follicle numbers decrease significantly during the last years of reproductive life, however, a restricted amount of inactive primordial follicles remain in the ovary during the first stages of menopause [34]. Approximately one thousand primordial follicles are estimatedto be present in the ovaries during menopause [35]. As a result, a percentage of patients with ovarian insufficiency under HT may report natural ovulation and conception [36] but nonetheless, such an event is very unlikely to occur [37]. The fact that our patient experienced menstrual cycle restoration 6 weeks following PRP treatment is impressive and directly links the two events, namely PRP treatment and folliculogenesis through the association of cause and effect. Ovarian rejuvenation in this case could not be interpreted as a random event related to HT. This is reinforced by the fact that the patient abandoned HT 4 months prior to PRP application, minimizing the possibility of attributing the results to HT or another unlikely random factor. This fact strengthens the deduction that the restoration of menstruation and pregnancy were directly associated with and enabled by PRP. 

It may be important to note that no adverse side effects were reported following treatment. In fact, the patient described the PRP treatment as a ‘psychological boost’ towards her perception of her reproductive status. PRP may enable the “hope” of a positive outcome in premature menopause, which is commonly accepted as a final scenario. Nonetheless, the patients’ perception and psychological response with regards to the final outcome -being positive or negative-may be viewed as both sides of the same coin. Introducing the possibility of restoring ovarian functionality may be clinically powerful, and extends to various levels, from the psychological to the bioethical. IVF has revolutionized the world not only with regards to medical aspects, but also from a social and bioethical standpoint. Making assisted reproductive techniques accessible to women facing ovarian failure—perhaps through PRP treatment—raises an array of profound questions and dilemmas. Should future clinical trials find PRP to be effective—as it is anticipated—the next step should be the careful implementation of criteria regarding use, considering the overall well-being of patients, especially from a social and bioethical standpoint. 

Undoubtedly, dilemmas and many questions will be raised regarding the components involved in shaping PRP treatment, especially since it seems to offer the possibility of at least a short-term solution to premature menopause, thus setting the basis for the restoration of fertility. How did PRP treatment enable an IVF cycle in a prematurely menopausal patient? To what extent is it efficient and safe to use? Could it contribute towards high-risk pregnancies, an increased probability of spontaneous abortions, or futile failed attempts affecting routine clinical practice? These are valid concerns that merit investigation. If further proof of the application of PRP is presented in the future, validated by the anticipated large clinical trials, will it be safe to extrapolate that it could become an option for prematurely menopausal women aiming to achieve a pregnancy? 

This case report cannot serve as the answer to all the questions raised. It does, however, set the basis for further research, data, and evidence regarding PRP implementation in clinical practice. This is the first report on a prematurely menopausal woman with irreversible ovarian dysfunction achieving a biochemical pregnancy employing her own oocytes. The horizontal implementation of this procedure entails major considerations as it is and constitutes—without a doubt—a biological paradox.
